# Regression of conjunctival tumor during dietary treatment of celiac disease

**DOI:** 10.4103/0301-4738.67071

**Published:** 2010

**Authors:** Samuray Tuncer, Baris Yeniad, Gonul Peksayar

**Affiliations:** Istanbul University, Istanbul Faculty of Medicine, Department of Ophthalmology, Ocular Oncology Service, Capa 34390, Istanbul, Turkey

**Keywords:** Celiac disease, conjunctiva, gluten-free diet, Kaposi sarcoma

## Abstract

A 3-year-old girl presented with a hemorrhagic conjunctival lesion in the right eye. The medical history revealed premature cessation of breast feeding, intolerance to the ingestion of baby foods, anorexia, and abdominal distention. Prior to her referral, endoscopic small intestinal biopsy had been carried out under general anesthesia with a possible diagnosis of Celiac Disease (CD). Her parents did not want their child to undergo general anesthesia for the second time for the excisional biopsy. We decided to follow the patient until all systemic investigations were concluded. In evaluation, the case was diagnosed with CD and the conjunctival tumor showed complete regression during gluten-free dietary treatment. The clinical fleshy appearance of the lesion with spider-like vascular extensions and subconjunctival hemorrhagic spots, possible association with an acquired immune system dysfunction due to CD, and spontaneous regression by a gluten-free diet led us to make a presumed diagnosis of conjunctival Kaposi sarcoma.

Celiac disease (CD) is an autoimmune gluten-induced enteropathy that typically causes intestinal malabsorption syndrome in childhood. The clinical course may be complicated by extra-intestinal immunological disorders such as dermatitis herpetiformis, cutaneous vasculitis, polyarteritis, glomerulonephritis, chronic liver disease, polymyositis, and myocarditis.[1] Although rare, ocular involvement has been reported as well.[2–4] These include immune-complex mediated uveitis,[2] ocular myopathy,[3] and Vogt-Koyanagi-Harada disease.[4] We report herein a unique case with a very unusual conjunctival tumor that showed complete regression during dietary treatment of CD.

## Case Report

A 3-year-old girl presented with a complaint of hemorrhagic tear episodes of 3 months duration in the right eye. Her parents stated that the reddish conjunctival lesion was detected elsewhere one month ago and was unresponsive to topical steroids, and hence, excisional biopsy was suggested. The patient was referred to our clinic to get a second opinion.

Her past medical history revealed premature cessation of breast feeding, intolerance to the ingestion of baby foods, anorexia, and abdominal distention since 2 months. Her weight and height percentiles were subnormal compared to her age group. From 26 months of age, she had recurrent serous otitis media treated with systemic antibiotics. However, the primary etiology could not be determined by her pediatrician in the first 3 years of life.

**Figure 1:**
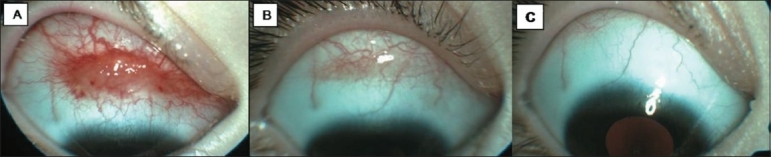
A 3-year-old girl, who had a 3-months history of hemorrhagic tear episodes, presented with a painless and reddish conjunctival lesion in the right eye. (A) Anterior segment photograph of the right eye showing reddish, fleshy, and highly vascular spider-like lesion on the superior bulbar conjunctiva. (B) After one week of follow-up with a gluten-free diet, spontaneous regression of the conjunctival lesion was noted. (C) After 2 months of follow-up, the conjunctival lesion disappeared completely

Our initial visit showed that the visual acuities were 20/20 in both eyes. Slit-lamp examination of the right eye revealed a reddish, elevated, and highly vascular spider-like lesion on the superior bulbar conjunctiva, measuring 12×4×2 mm [Fig. 1A]. Our presumed diagnosis was conjunctival Kaposi sarcoma (KS). Prior to her referral, endoscopic small intestinal biopsy had been carried out under general anesthesia with a possible diagnosis of CD in another hospital. Therefore, her parents did not want their child to undergo general anesthesia for the second time for the excisional biopsy. We decided to follow the patient without any intervention until all systemic investigations were concluded.

The blood test for HIV antibody was negative. Serology showed high anti-gliadin and anti-endomysial immunoglobulin A antibody levels. Endoscopic intestinal biopsy demonstrated partial villous atrophy, intraepithelial lymphocytosis, and crypt hyperplasia consistent with CD. Genetic testing of the family members revealed high maternal autoantibody titers for CD.

After the diagnosis of CD, gluten-free diet was instituted. The conjunctival lesion gradually regressed [Fig. 1B] and disappeared completely after 2 months [Fig. 1C]. She was completely asymptomatic and the conjunctival lesion did not recur after 9 months of follow-up.

## Discussion

CD is an autoimmune small bowel enteropathy due to an immune-mediated hypersensitivity to gluten.[5] It has been associated with type I diabetes mellitus and other extraintestinal autoimmune disorders.[4,5] These autoimmune disorders generally respond favorably to a gluten-free diet.[5]

Ocular involvement in CD has been reported in a very few number of case reports.[2–4] To our knowledge, conjunctival involvement during the course of CD has not been reported previously in the literature. The pathophysiology underlying this situation remains unknown. However, under the light of the literature regarding ocular involvement of CD,[2–4] autoimmune mechanisms and immunogenetic factors should play a role in the pathogenesis. In our case, we speculate that the growth of this conjunctival lesion occurred due to the anti-gliadin antibodies and/or circulating immune complexes that have deposited in the conjunctiva causing an arrangement of capillary channels.

In our case, the differential diagnoses of such a conjunctival lesion includes KS, subconjunctival hemorrhage, malignant melanoma, squamous cell carcinoma, pyogenic granuloma, cavernous hemangioma, lymphoma, caroticocavernous fistula, foreign body granuloma, and lymphangioma.[7] We did not perform incisional or excisional biopsy because the parents did not allow us to perform such a procedure under general anesthesia. However, the clinical fleshy appearance of the lesion with numerous spider-like vascular extensions and minute subconjunctival hemorrhagic spots, possible association with an acquired immune system dysfunction due to CD, and spontaneous regression by a gluten-free diet led us to make a presumed diagnosis of conjunctival KS. In the literature, spontaneous resolution of cutaneous KS was noted after withdrawal of corticosteroids in a patient who had been treated for pemphigus.[8]

Acquired defects of the immune system have been found in patients with CD and KS. Thus, concurrent presence of both disorders may be observed. There is only one case report that presented histopathology-proven visceral KS in a 54-year-old man with CD.[6]

In conclusion, we present a very unusual conjunctival tumor in a patient with CD that showed complete regression by a gluten-free diet. The precise pathological nature of this conjunctival lesion remains unknown due to the lack of histopathological confirmation. However, prompt regression of the conjunctival lesion during gluten-free diet suggests a possible relationship to CD and an autoimmune process.
